# Perinatal and Socio-Demographic Factors Associated With Autism Spectrum Disorder in Children Aged Three to Six Years: A Hospital-Based Cross-Sectional Study

**DOI:** 10.7759/cureus.109065

**Published:** 2026-05-17

**Authors:** Rojalin Rout, Pravati Jena, Manas Ranjan Behera, Sanjay Kumar Sahu

**Affiliations:** 1 Paediatrics, Kalinga Institute of Medical Sciences, Bhubaneswar, IND

**Keywords:** associated factors, autism spectrum disorder, maternal smoking, perinatal factors, socio-economic factors

## Abstract

Background: Autism spectrum disorder (ASD) is a neurodevelopmental disorder that is being identified with increasing frequency worldwide. It arises from a complex interplay of genetic, environmental, perinatal, and socio-demographic influences. A better understanding of these contributing factors is important for improving early identification and guiding preventive strategies. Within this framework, the present study sought to evaluate the perinatal and socio-demographic factors linked to ASD in children aged three to six years.

Methods: This cross-sectional study, conducted in a hospital setting, took place at a tertiary care center in Bhubaneswar, Odisha, India, between June 2024 and March 2025. A total of 160 children aged three to six years with suspected ASD were included using convenience sampling. Screening was performed using the Trivandrum Autism Behavioural Checklist (TABC), followed by confirmatory diagnosis with the INCLEN Diagnostic Tool for Autism Spectrum Disorder (INDT-ASD). Data on perinatal factors (gestational age, birth order, mode of delivery, and pregnancy-related complications, including maternal anemia, gestational diabetes, pregnancy-induced hypertension, hypothyroidism, premature rupture of membranes, antepartum hemorrhage, and meconium-stained amniotic fluid) and socio-demographic variables (age, sex, family type, and maternal smoking) were collected. Categorical variables were analyzed using the chi-square or Fisher's exact test, while continuous variables were compared using the independent samples t-test. Odds ratios (OR) with 95% confidence intervals (CI) were computed, and a p-value < 0.05 was taken as statistically significant.

Results: Out of 160 children aged three to six years with suspected ASD, 25 (15.6%) were diagnosed with ASD. A significant male preponderance was noted (OR: 3.61; 95% CI: 1.17-11.10; p = 0.026). Maternal anemia (OR: 3.35; 95% CI: 1.39-8.05; p = 0.007) and maternal smoking (OR: 10.22; 95% CI: 3.97-26.29; p < 0.001) were significantly associated with ASD. Children from nuclear families had significantly lower odds of ASD compared to joint families (OR: 0.09; 95% CI: 0.04-0.24; p < 0.001). No other factors showed statistically significant associations.

Conclusion: Male gender, maternal anemia, smoking, and family structure were identified as important associated factors. These observations highlight the need to address modifiable maternal risk factors and consider socio-cultural influences in early detection. Further large-scale prospective studies are needed to confirm these associations.

## Introduction

Autism spectrum disorder (ASD) is a multifaceted neurodevelopmental disorder marked by challenges in social interaction, difficulties in communication, and the presence of restricted or repetitive behaviours [1,2]. In recent decades, there has been an increase in prevalence globally due to increased awareness and possible changes in underlying risk patterns [3]. It has become an important public health concern, not only because of its rising prevalence but also due to its profound emotional and social impact on affected children and their families. The causes of ASD are multifactorial, arising from a complex interplay between genetic predisposition and environmental factors. Genetic factors contribute significantly, with evidence suggesting involvement of multiple genes affecting synaptic function and neural connectivity [4-6]. However, genetic predisposition alone does not fully explain the variability in clinical presentation, highlighting the role of additional contributing factors.

Environmental and perinatal factors have gained increasing attention for their potential role in early brain development. Maternal conditions, such as anemia, may lead to reduced oxygen delivery and altered neurodevelopment, while exposure to tobacco smoke during pregnancy can affect neuronal signaling and synaptogenesis [7-9]. Obstetric factors, including preterm birth, complications during pregnancy, and perinatal stress, have also been investigated, although findings remain inconsistent across studies.

Socio-demographic factors, including family structure, parental characteristics, and caregiving environment, may further influence developmental trajectories. In culturally diverse settings, variations in family dynamics and early stimulation may contribute to differences in ASD identification and progression [10-13].

Importantly, ASD does not stem from a single cause but instead reflects the combined impact of multiple interacting factors. The interplay between genetic vulnerability and environmental exposures, particularly during critical periods of brain development, may alter neural pathways and contribute to the onset and progression of ASD. Understanding these interactions is essential for early identification and for developing context-specific preventive strategies.

Despite growing evidence globally, data from developing regions remain limited. Accordingly, this study was conducted in a tertiary care setting to evaluate perinatal and socio-demographic factors associated with ASD in children aged three to six years.

## Materials and methods

Study design and setting

This hospital-based, cross-sectional study was conducted in the Department of Pediatrics at a tertiary care center in eastern India from June 2024 to March 2025. The objective was to evaluate perinatal and socio-demographic factors associated with ASD in children aged three to six years.

Study population

Children aged three to six years presenting with clinical suspicion of ASD during the study period were enrolled. Inclusion criteria comprised children suspected of having ASD. Children with global developmental delay, inborn errors of metabolism, extreme prematurity (< 28 weeks of gestation), and known fetal or chromosomal anomalies were excluded.

Sample size and sampling technique

Given the exploratory nature of the study, a formal sample size calculation was not undertaken. To ensure comprehensive data capture, all eligible children presenting during the study period were included in the study. A total of 160 children were included in the study. Convenience sampling was adopted, and all eligible children attending the hospital during the study period were recruited.

Data collection and diagnostic assessment

After obtaining written informed consent from parents or guardians, data were collected using a predesigned structured proforma. Information on perinatal factors (gestational age, mode of delivery, birth asphyxia, maternal conditions, such as anemia, gestational diabetes, hypertension, and drug exposure) and socio-demographic variables (age, sex, parental age, education, occupation, socioeconomic status, family type, family history, and comorbidities, such as attention-deficit/hyperactivity disorder, ADHD) was recorded.

Screening for ASD was initially performed using the Trivandrum Autism Behavioural Checklist (TABC) [14]. Children who screened positive underwent confirmatory diagnosis using the INCLEN Diagnostic Tool for Autism Spectrum Disorder (INDT-ASD), a validated tool for Indian children aged two to nine years [15]. Diagnosis was based on the Diagnostic and Statistical Manual of Mental Disorders, Fourth Edition, Text Revision (DSM-IV-TR) criteria. The INDT-ASD evaluates three domains - social interaction, communication, and restricted or repetitive behaviours - using a binary scoring system. Each assessment was conducted by trained professionals and required approximately 30-45 minutes.

Statistical analysis

Data were recorded in a Microsoft Excel (Microsoft® Corp., Redmond, WA) spreadsheet and analyzed using Statistical Product and Service Solutions (SPSS, version 27.0; IBM SPSS Statistics for Windows, Armonk, NY). Continuous variables were summarized as mean ± standard deviation, whereas categorical variables were reported as frequencies and percentages. Normality was assessed using the Shapiro-Wilk test. Continuous variables were analyzed using the independent samples t-test, while categorical variables were assessed using the chi-square test. Multivariate analysis could not be performed due to the limited sample size and number of outcome events. Therefore, findings are based on bivariate analysis and should be interpreted with caution. A p-value of less than 0.05 was considered statistically significant.

Ethical considerations

Ethical approval for the study was obtained from the Institutional Ethics Committee of the Kalinga Institute of Medical Sciences, Bhubaneswar (Ref. No. KIIT/KIMS/IEC/1919/2024). Written informed consent was secured from the caregivers of all participants. Participant confidentiality and anonymity were rigorously upheld, and the data were utilized exclusively for academic purposes.

## Results

Out of 160 children aged three to six years evaluated, 25 (15.6%) were diagnosed with ASD. The mean age of participants was 4.34 ± 0.86 years. No statistically significant differences were observed between the ASD and non-ASD groups in terms of age, birth weight, or parental age (p > 0.05).

A significant male predominance was noted, with boys showing greater odds of ASD than girls (OR: 3.61; 95% CI: 1.17-11.10; p = 0.026).

Among maternal factors, anemia during pregnancy was significantly associated with ASD (OR: 3.35; 95% CI: 1.39-8.05; p = 0.007). Maternal smoking also showed a strong association with ASD (OR: 10.22; 95% CI: 3.97-26.29; p < 0.001).

Children from nuclear families had significantly lower odds of ASD compared to joint families (OR: 0.09; 95% CI: 0.04-0.24; p < 0.001). However, this finding should be interpreted with caution in the given sample size. These findings are summarized in Table 1.

**Table 1:** Association of socio-demographic and selected maternal factors with ASD (OR, 95% CI, and p-value) Values are expressed as numbers. The chi-square test was used for categorical variables; Fisher's exact test was applied where appropriate. A p-value < 0.05 was considered statistically significant. Variables with zero cell counts may yield unstable estimates; therefore, odds ratios were not calculated. OR: Odds Ratio; CI: Confidence Interval; ASD: Autism Spectrum Disorder, Ref.: Reference

Variables	Category	No ASD (n=135)	ASD (n=25)	χ² (chi-square)	P value	OR	95% CI
Gender	Female	55	4	5.00	0.026	Ref.	-
	Male	80	21			3.61	1.17-11.10
Maternal anemia	No	102	12	7.31	0.007	Ref.	-
	Yes	33	13			3.35	1.39-8.05
Smoking	No	115	9	26.54	<0.001	Ref.	-
	Yes	20	16			10.22	3.97-26.29
Psychiatric illness	No	123	25	Fisher's exact test	0.120	-	-
	Yes	12	0			-	-
Family size	Joint	19	16	28.97	<0.001	Ref.	-
	Nuclear	116	9			0.09	0.04-0.24

Other perinatal factors, including gestational age, birth order, mode of delivery, and pregnancy-related complications (e.g., gestational diabetes mellitus, pregnancy-induced hypertension, hypothyroidism, premature rupture of membranes, and antepartum hemorrhage), were not significantly associated with ASD (p > 0.05). Meconium-stained amniotic fluid showed a trend toward association but did not reach statistical significance (p = 0.082). These findings are presented in Table 2.

**Table 2:** Association of perinatal factors with ASD (OR, 95% CI, and p-value) Values are expressed as numbers. The chi-square test was used for categorical variables; Fisher's exact test was applied where appropriate. For variables with more than two categories (e.g., birth order), odds ratios were calculated using the first category as reference. A p-value < 0.05 was considered statistically significant. OR: Odds Ratio; CI: Confidence Interval; ASD: Autism Spectrum Disorder; GDM: Gestational Diabetes Mellitus; PIH: Pregnancy-Induced Hypertension; PROM: Premature Rupture of Membranes; APH: Antepartum Hemorrhage, Ref.: Reference

Variables	Category	No ASD (n=135)	ASD (n=25)	χ² (chi-square)	P value	OR	95% CI
Birth order	1	81	19	2.87	0.238	Ref.	-
	2	37	3			0.35	0.09-1.30
	3	17	3			0.76	0.18-3.22
Gestational age	<37 weeks	73	17	1.67	0.196	Ref.	-
	≥37 weeks	62	8			0.55	0.22-1.37
Pregnancy outcome	Single	123	21	Fisher's exact test	0.283	Ref.	-
	Twin	12	4			1.95	0.57-6.63
Mode of delivery	LSCS	97	19	0.18	0.672	Ref.	-
	VD	38	6			0.81	0.30-2.17
Meconium	No	108	16	3.03	0.082	Ref.	-
	Yes	27	9			2.25	0.90-5.64
PROM	No	125	23	Fisher's exact test	0.914	Ref.	-
	Yes	10	2			1.09	0.22-5.29
GDM	No	91	19	0.73	0.315	Ref.	-
	Yes	44	6			0.65	0.24-1.75
PIH	No	77	13	0.22	0.639	Ref.	-
	Yes	58	12			1.23	0.52-2.88
APH	No	125	23	Fisher's exact test	0.914	Ref.	-
	Yes	10	2			1.09	0.22-5.29
Hypothyroidism	No	107	17	1.52	0.218	Ref.	-
	Yes	28	8			1.80	0.70-4.59

No significant association was observed for psychiatric illness; however, interpretation is limited due to small subgroup numbers. These findings should be interpreted with caution due to the exploratory nature of the study.

## Discussion

The present study examined perinatal and socio-demographic factors associated with ASD in children aged three to six years and supports the understanding that ASD is influenced by multiple interacting factors rather than a single cause.

A clear male predominance was observed, which is consistent with findings from previous studies reporting higher ASD prevalence among males [16]. Although the exact mechanisms remain unclear, genetic susceptibility and hormonal influences have been proposed as possible explanations [17].

Maternal anemia during pregnancy was significantly associated with ASD in this study. This finding may reflect the impact of maternal nutritional status on fetal brain development, particularly in settings where anemia is common [17,18]. Similarly, maternal smoking showed a strong association with ASD, supporting the role of prenatal environmental exposures in influencing neurodevelopment [19,20].

An important and somewhat divergent finding in the present study was the association between joint family structure and ASD. In contrast, studies from high-income countries have more often linked nuclear family settings or reduced social engagement with developmental concerns. The difference observed here may reflect sociocultural variations in caregiving practices, family dynamics, and early childhood stimulation. In extended family systems, variations in caregiving patterns and levels of individualized parent-child interaction may influence early developmental experiences, although this remains speculative and warrants further investigation in larger and more diverse populations [10]. However, this finding should be interpreted cautiously, as it may be influenced by unmeasured confounders and the relatively small sample size.

Although not included in the primary analysis table, an exploratory observation in this study suggested a higher proportion of ASD among children of mothers engaged in high-stress professions. However, this finding should be interpreted with caution, as detailed quantification of occupational stress and potential confounders was not performed. Previous studies have suggested that maternal stress during pregnancy may influence fetal neurodevelopment through hormonal and inflammatory pathways, but the evidence remains inconclusive [21,22]. Further studies with more robust designs are required to explore this relationship.

In contrast, several perinatal factors traditionally linked with ASD, such as gestational age, mode of delivery, and common pregnancy-related complications, did not show significant associations in this study. These findings are in line with some studies but differ from others, reflecting the heterogeneity of ASD and the influence of population-specific factors.

Strengths and limitations

This study provides insight into ASD-associated factors in an Indian tertiary care setting using a validated diagnostic tool. However, limitations include the relatively small sample size, hospital-based design, and reliance on bivariate analysis. It is important to note that some estimates, particularly those based on small subgroup numbers, may be unstable. The cross-sectional design of the study limits causal inference, and the absence of multivariate analysis restricts adjustment for potential confounders. Additionally, genetic and detailed environmental factors were not assessed. Therefore, context-specific research in low- and middle-income settings is essential.

## Conclusions

This study highlights that ASD is influenced by a combination of maternal and socio-demographic factors rather than a single cause. Male gender, maternal anemia, and maternal smoking showed significant associations with ASD, while family structure also appeared to play a role. These findings are partly consistent with global evidence, but they also reflect context-specific influences relevant to the local population. The results emphasize the importance of addressing modifiable maternal factors, particularly during pregnancy, as part of early preventive strategies. At the same time, the observed associations should be interpreted with caution due to the study's cross-sectional design and limited sample size. Further large-scale, prospective studies are needed to better understand these relationships and to explore the interaction between biological and environmental factors in ASD.
